# Genomic prediction for fusiform rust disease incidence in a large cloned population of *Pinus taeda*

**DOI:** 10.1093/g3journal/jkab235

**Published:** 2021-07-22

**Authors:** Mohammad Nasir Shalizi, W Patrick Cumbie, Fikret Isik

**Affiliations:** 1 Department of Forestry and Environmental Resources, North Carolina State University, Raleigh, NC 27695-8002, USA; 2 ArborGen Inc., Ridgeville, SC 29472, USA

**Keywords:** genomic selection, cloning, categorical traits, loblolly pine, Genetic relatedness, Bayesian whole genome regression, SNP markers, Cronartium quercuum f. sp. fusiforme

## Abstract

In this study, 723 *Pinus taeda* L. (loblolly pine) clonal varieties genotyped with 16920 SNP markers were used to evaluate genomic selection for fusiform rust disease caused by the fungus *Cronartium quercuum* f. sp*. fusiforme*. The 723 clonal varieties were from five full-sib families. They were a subset of a larger population (1831 clonal varieties), field-tested across 26 locations in the southeast US. Ridge regression, Bayes B, and Bayes Cπ models were implemented to study marker-trait associations and estimate predictive ability for selection. A cross-validation scenario based on a random sampling of 80% of the clonal varieties for the model building had higher (0.71–0.76) prediction accuracies of genomic estimated breeding values compared with *family* and *within-family* cross-validation scenarios. Random sampling within families for model training to predict genomic estimated breeding values of the remaining progenies within each family produced accuracies between 0.38 and 0.66. Using four families out of five for model training was not successful. The results showed the importance of genetic relatedness between the training and validation sets. Bayesian whole-genome regression models detected three QTL with large effects on the disease outcome, explaining 54% of the genetic variation in the trait. The significance of QTL was validated with GWAS while accounting for the population structure and polygenic effect. The odds of disease incidence for heterozygous AB genotypes were 10.7 and 12.1 times greater than the homozygous AA genotypes for SNP11965 and SNP6347 loci, respectively. Genomic selection for fusiform rust disease incidence could be effective in *P. taeda* breeding. Markers with large effects could be fit as fixed covariates to increase the prediction accuracies, provided that their effects are validated further.

## Introduction

Threshold traits (dichotomous or polychotomous characters) are discrete variables when assessed in genetic studies (Lynch and Walsh 1998). Dichotomous traits have two levels of phenotypic classes; “affected (yes)” and “not-affected (no).” Polychotomous traits can have more than two levels of discrete phenotypic classes. Some threshold traits behave like continuous traits and are not inherited in a simple Mendelian manner (Falconer and Mackay 1996). These traits can be heritable and are affected by genetics and the environment. For genetic analyses, threshold traits are sometimes transformed to continuous variables, known as liability, to estimate variance components (Falconer and Mackay 1996). Generalized linear models with various link functions have been used for the analysis of discrete variables (Nelder and Wedderburn 1972; Gilmour *et al.* 1985).

In pine species of the southern US, fusiform rust disease caused by *Cronartium quercuum* f. sp. *fusiforme* is usually assessed as a discrete dichotomous response. The pathogen has co-evolved with the host pine species in the region over millions of years (Kubisiak *et al.* 2005). The disease is an important problem in *Pinus taeda* L. plantations in the southern US. The fungus causes damage to more than 100 million US dollars annually in young pine plantations (Cubbage *et al.* 2000). The disease suppresses growth, reduces wood quality, and increases mortality in pine plantations in the region.

Numerous studies suggested that fusiform rust disease in *P. taeda* is controlled by a few major quantitative trait loci (QTL) named *Fr* genes. A resistant single dominant locus, *Fr1*, was mapped by association of the phenotype with random amplified polymorphic (RAPD) DNA genetic markers in four specific families of *P. taeda* (Wilcox *et al.* 1996). Using the same method, additional loci were discovered in different *P. taeda* families (Amerson *et al.* 2015). In the above studies, the QTL could be detected when the progeny of parents were challenged with specific spore isolates of the pathogen.

In recent years, single nucleotide polymorphisms (SNPs) markers were used in GWAS to map QTL and explore genomic selection for fusiform rust disease. In two independent clonally replicated *P. taeda* populations, five SNP markers accounted for 30% (Quesada *et al.* 2014) and 45% of the total genetic variance for disease incidence (Cumbie *et al.* 2020). These two studies used different sets of SNP markers. In a most recent study, Lauer and Isik (2021) mapped three major QTL affecting fusiform rust disease in two *P. taeda* full-sib crosses. Their results showed that progeny of one cross were immune to the disease when they had two QTL. In *P. taeda* breeding in the southern US, none of the QTLs have been employed because the marker-tagged QTLs were specific to individual pine families.

In practical breeding of *P. taeda* in the southern US, the disease incidence is assessed as a binary outcome and is analyzed as a continuous trait on liability scale (Isik *et al.* 2008). Numerous studies suggested that selection for fusiform rust disease resistance at the family level is effective in *P. taeda* because of high repeatability of family means (McKeand *et al.* 1999; Isik *et al.* 2003, 2004, 2008; Cumbie *et al.* 2011). This is not the case for within-family selection because of low within-family heritability estimates. Cloned progeny testing and selection have been proposed to increase within-family selection efficiency for the species (Foster and Shaw 1988; Isik *et al.* 2003, 2005; McKeand *et al.* 2003, 2006). However, clonal progeny testing is costly and adds another three years to the breeding cycle of *P. taeda*. Genomic selection may offer an alternative to clonal progeny testing for within-family selection (Isik 2014).

In this study, we investigated the efficiency of within-family genomic selection for *P. taeda*, the most economically important tree species in the US (McKeand 2019). Our study is based on a large, cloned progeny population (1831 clonal varieties, 723 were genotyped) that originated from five crosses. We were particularly interested in knowing if there are QTL controlling fusiform rust disease and explore genomic selection strategies for within-family selection. The results from this study may have large implications for conifer breeding for disease and pest incidence.

## Materials and methods

### Genetic material and experimental design

A total of 1831 clonal varieties from 37 full-sib families were cloned for within-family selection in a *P. taeda* population in the southeastern US. The number of clonal varieties (genotype) per family ranged between 1 and 238 with an average of 49. Clonal varieties were produced via somatic embryogenesis (Becwar *et al.* 1990), in which developing embryos were harvested from immature cones and brought into culture to produce multiple copies of the same genotype. Plantlets were raised in a greenhouse for 8–12 weeks and subsequently grown outdoors for 6 months prior to field trial establishment.

The study was established at 26 locations (sites) in the southeastern US. Each site consisted of six incomplete blocks with row-column configuration. Not all the trials were established in the same year because of logistics of producing plantlets. Trials were established in seven test series starting in 2006 and were completed in 2008. The number of tests established per series ranged between 2 and 6. Test sites were strongly connected within a test series (69–396 genotypes in common), whereas the overlap between tests across series was weak. The average number of trees (identical copies) per clonal variety was ∼17 trees across all the test series. The layout of the reps and number of crosses and clones differed among sites.

### Phenotyping and genotyping

About 31,411 trees (1831 clonal varieties × 17 copies each) were assessed for the incidence of fusiform rust disease (presence of galls or no galls) at age 6 years. For this study, 723 clonal varieties from five full-sib families were genotyped. Most of these families were related sharing a parent or a grandparent (Supplementary Figure S1). The genotyped varieties were distributed across all 26 trials. The number of clonal varieties per family ranged between 46 and 224. For genotyping, DNA was extracted from young needle tissues of trees. A proprietary 17 K custom SNP array was developed using the Applied Biosystems^TM^ Axiom^TM^ array from ThermoFisher Scientific. SNPs loci were initially screened with a 192 sample set of diverse genotypes from across the natural range of *P. taeda*. Candidate SNP sequences used in the screening were developed as a part of the PINEMAP project (Coordinated Agricultural Project funded by the USDA National Institute of Food and Agriculture, Award #2011-68002-30185). SNPs were selected for inclusion on the final array based on a call rate success and a minor allele frequency of 5%. Markers violating a nonzero minor allele frequency less than 0.02 and a lower than expected proportion of heterozygotes were discarded. Missing genotypes were imputed with LinkImpute software (Money *et al.* 2015). The software uses the LD-kNNi algorithm developed and modified by Troyanskaya *et al.* (2001) and Schwender (2012). The method imputes missing genotypes using normalized distance and *k* nearest neighbors between genotypes. The final number of markers used in the study was 16920.

### Statistical analysis

#### Variance components and heritability:

The incidence of fusiform rust disease of 1831 genotypes was analyzed using the following generalized linear mixed model.

(1) $$\eta =\log\left(\frac{p}{1-p}\right)=1\mu +Xs+Z_{1}b+Z_{2}c+Z_{3}r+Z_{4}a+Z_{5}n+e$$

where η is the logit link function g(μ) of the vector of response variable (incidence or no incidence); p is the probability of trait incidence; $\log\left(p/1-p\right)$ is the log of odds; μ is the conditional mean; X and Z are incidence matrices for the fixed and random effects respectively; s is the vector of site fixed effect (s=26); b is the vector for random replicate effect (b=6) nested within site with $b∼\mathrm{MVN}(0,I\sigma_{b}^{2})$; c is the vector of random column effect (c=120) nested within rep with $c∼\mathrm{MVN}(0,I\sigma_{c}^{2})$; r is the vector of random row effect (r=145) nested within rep with $r∼\mathrm{MVN}(0,I\sigma_{r}^{2})$; a is the vector of random additive genetic effects (a=1831) with $a∼\mathrm{MVN}(0,G_{a}⊗A)$; n is the vector of random nonadditive genetic effects (n=1831) with $n∼\mathrm{MVN}(0,I\sigma_{n}^{2})$; and e is the vector of random errors with $e∼\mathrm{MVN}\left(0,{I\sigma }_{e}^{2}\right)$. The symbol ⊗ is the direct (Kronecker) product operator, I is an identity matrix of its proper dimensions, $G_{a}$ is the s-by-s variance-covariance matrix of the genotype nested within environment effect, and A is the a-by-a numerator relationship matrix derived from pedigree.

Various variance-covariance structures were fit to the additive genetic effects (a), such as compound symmetry, heterogeneous variance structure, and factor analytics to model the additive genetic and genetic by environment interactions. Heterogeneous variance-covariance structure was the best fit. The mathematical form of heterogeneous $G_{a}$ structure is (Isik *et al.* 2017)

(2) $$G_{a}⊗A=\left(\begin{array}{cc} \begin{array}{cc} \sigma_{ge1}^{2} & \sigma_{ge1}\sigma_{ge2}\rho \\ \sigma_{ge1}\sigma_{ge2}\rho & \sigma_{ge2}^{2} \end{array} & \begin{array}{cc} \cdots & \sigma_{ge1}\sigma_{ge26}\rho \\ \cdots & \vdots \end{array}\\ \begin{array}{cc} \vdots & \cdots \\ \sigma_{ge1}\sigma_{ge26}\rho & \cdots \end{array} & \begin{array}{cc} \ddots & \sigma_{ge25}\sigma_{ge26}\rho \\ \sigma_{ge25}\sigma_{ge26}\rho & \sigma_{ge26}^{2} \end{array} \end{array}\right)⊗A$$

where the diagonal elements are site-specific additive genetic variances and off-diagonal elements are covariance between pairs of sites. This covariance structure provided a uniform correlation among site pairs.

A simple IID (independent and identically distributed) structure was fitted for nonadditive genetic effects in the model. This structure provides a single constant nonadditive genetic variance across all sites but no covariance between pairs of sites. Nonadditive genetic by environment interaction was not significant. Residual log-likelihood ratio test was used to assess the significance of additive and nonadditive genetic variances.

Using the observed variance components from the above model, clone-mean narrow-sense, and broad-sense heritabilities were estimated using the derivations according to Isik *et al.* (2017):

(3) $$h_{\bar{c}}^{2}=\frac{r_{a}{\bar{\sigma }}_{a}^{2}}{\frac{{\bar{\sigma }}_{a}^{2}}{s}+\frac{(s-1)r_{a}{\bar{\sigma }}_{a}^{2}}{s}+\frac{(s-1)\sigma_{n}^{2}}{s}+\frac{\sigma_{e}^{2}}{n_{h}}}$$

(4) $$H_{\bar{c}}^{2}=\frac{r_{a}{\bar{\sigma }}_{a}^{2}+\sigma_{n}^{2}}{\frac{{\bar{\sigma }}_{a}^{2}}{s}+\frac{(s-1)r_{a}{\bar{\sigma }}_{a}^{2}}{s}+\frac{(s-1)\sigma_{n}^{2}}{s}+\frac{\sigma_{e}^{2}}{n_{h}}}$$

where ${\bar{\sigma }}_{a}^{2}$ is the average square root of product of pairwise additive genetic variances within environments; $\sigma_{n}^{2}$ is nonadditive genetic variance; $\sigma_{e}^{2}$ is the residual variance; $r_{a}$ is the additive genetic correlation between pairs of sites, s is the number of sites (s=26), and $n_{h}$ is the harmonic mean number of trees per clonal variety ($n_{h}=11.657$). $\sigma_{e}^{2}$ is the variance of standard binomial distribution $\pi^{2}/3\;=\;3.29$ (Gilmour *et al.* 1985). Standard errors of heritabilities were approximated using the delta method (Lynch and Walsh 1998). The models were run using ASReml software version 4.1 (Gilmour *et al.* 2015). Variance components and heritability estimates were calculated using a pin file in the ASReml software.

#### Estimation of pseudo-phenotypes for genomic selection

The solutions from Equation (1) for the random clonal variety effects are the best linear unbiased predictions (BLUPs). These predictions may not be suitable as pseudo-phenotypes for genomic prediction models due to shrinkage of estimates toward the mean. Because of shrinkage, they have a smaller variance. Also, the use of pedigree incorporates parental effects to estimate breeding values of trees which need to be removed (Garrick *et al.* 2009). We fit the following generalized linear mixed model to obtain the best linear unbiased estimates (BLUEs) of clonal variety effects to use as pseudo phenotype in genomic prediction models below.

(5) $$\eta =\log\left(\frac{p}{1-p}\right)=1\mu +X_{1}s+X_{2}g+Z_{1}b+Z_{2}c+Z_{3}r+e$$

In the model, the g is the vector of clonal variety fixed effect, $X_{2}$ is the design matrix. The nonadditive genetic effect in Equation (1) was dropped in this model. The experimental design factors row (*r*), column (*c*) and blocking (*b*) effects were fit as random factors, with the same assumptions as in Equation (1). Similarly, the vector of random errors had the expectations of $e\sim NID\left(0,{I\sigma }_{e}^{2}\right)$. The solutions for the fixed clonal variety effect are BLUE obtained by using the “predict” statement in ASReml software. The BLUE of clonal varieties from the model were used as pseudo-phenotypes to estimate marker-trait associations in whole-genome regression models. These estimates are adjusted for experimental design factors but are not shrunk toward the mean.

#### Expected and realized genomic relationships

The additive genetic relationships were calculated using the pedigree. Realized genomic relationship matrix was calculated using the regression method according to VanRaden (2008). where M is nxm matrix of SNP markers with elements −1, 0, and 1 for AA, AB, and BB genotypes, respectively; $g_{0}$ is the intercept, $g_{1}$ is the regression slope, 1 is the matrix of ones, A is the expected genetic relationship matrix calculated from pedigree, and E is the matrix of Mendelian sampling variation plus measurement error. In this method, the product of the M matrix with its transpose ($MM^{'}$**)** is regressed on A. The intercept ($g_{0}$) and the slope ($g_{1}$) were calculated by solving the following equation.

$MM^{'}=g_{0}11^{'}+g_{1}A+E$ (6)

$\left|\begin{array}{cc} n^{2} & \Sigma_{j}\Sigma_{k}A_{jk}\\ \Sigma_{j}\Sigma_{k}A_{jk} & \Sigma_{j}\Sigma_{k}A_{jk}^{2} \end{array}\right|\left|\begin{array}{c} g_{0}\\ g_{1} \end{array}\right|=\left|\begin{array}{c} \Sigma_{j}\Sigma_{k}{\left(MM^{'}\right)}_{jk}\\ \Sigma_{j}\Sigma_{k}{\left(MM^{'}\right)}_{jk}A_{jk} \end{array}\right|$ (7) 

The intercept and the slope estimated from Equation (6) were used to calculate the realized genomic relationship matrix as 

$G=\frac{MM^{'}-g_{0}11^{'}}{g_{1}}$ (8) 

The relationship matrices were calculated using the AGHmatrix package in R statistical software (Amadeu *et al.* 2016). Using the A and G matrices, average expected and realized relationship coefficients were calculated for individual families and family pairs.

#### Linkage disequilibrium

The homozygous and heterozygous genotype frequencies across all SNP loci were 0.813 (AA), 0.178 (AB), and 0.009 (BB). Minor allele frequency ranged between 0 and 0.50 with a mean of 0.10. The marker matrix was used to identify clusters of genotypes into families in order to better understand the structure in the population. Spectral decomposition was performed on the marker matrix using principal component analysis in R statistical software (R Core Team 2018). Heatmaps were generated to compare the expected additive genetic relationships (based on pedigree) and realized additive genetic relationship (based on SNP markers). Linkage disequilibrium among the three most significant and six other high ranking loci based on Bayesian whole-genome regression models was calculated as $r^{2}={{(h}_{AB}h_{ab}-h_{Ab}h_{bB})}^{2}/p_{A}p_{a}p_{B}p_{b},$ where $h_{AB},h_{ab},h_{Ab},h_{bB}$ are probability of observing gamete frequencies and p indicates frequency for the two alleles at loci A and B (Isik *et al.* 2017) (see Supplementary Figure S2). For construction of heatmaps the R package ggplot2 was used (Wickham 2016).

#### Marker-trait associations and genomic prediction

We fit the following whole-genome regression model to estimate genomic estimated breeding values (GEBV) and SNP marker effects

y=1μ+Wβ+e (9)

where y is the vector of best linear unbiased estimates (pseudo-phenotypes) obtained by solving mixed model equations in model Equation (5), μ is the overall mean, β is the vector of random marker effects, W is the incidence matrix of markers, e is the vector of random residual effects with $e∼\mathrm{NID}(0,I\sigma_{e}^{2})$, and 1 is the vector of ones.

Ridge regression implementation of Equation (9) (Hoerl and Kennard 1970) assumes multivariate normal prior distribution with mean zero and common variance for marker effects, $\beta ∼\mathrm{NID}\left(0,I\sigma_{\beta }^{2}\right)$. The model shrinks marker effects uniformly. This method estimates marker effects by minimizing residual sums of squares (Tibshirani 1996).

(10) $${\hat{\beta }}_{RR}=\mathrm{argmin}\left\{{\left|y-W\beta \right|}^{2}+\sigma_{\beta }^{2}\sum_{i=1}^{p}\beta_{i}^{2}\right\}$$

where, ${\hat{\beta }}_{RR}$ is the estimated marker effect and $\sigma_{\beta }^{2}$ is the regularization parameter. Other terms were explained before.

In the literature, there are some empirical data reporting large QTL effects on fusiform rust disease incidence in *P. taeda* (Amerson *et al.* 2015). To test this hypothesis, Bayes B model was fit, which assumes that each marker has a unique variance following a scaled inverse $\chi^{2}$ prior with two hyper parameters, $v_{\beta }$ and $S_{\beta }^{2}$, where $v_{\beta }$ are the degrees of freedom and $S_{\beta }^{2}$ is the scale parameter (Meuwissen *et al.* 2001; Gianola *et al.* 2009). The effect of each marker is fit with probability (1-π) which follows a univariate student’s *t*-distribution, $t(0,v_{\beta },S_{\beta }^{2})$ with degrees of freedom $v_{\beta }=5$ (BGLR default) and scale parameter $S_{\beta }^{2}∼\mathrm{gamma}$ (BGLR default), where *π* represents the probability of markers having zero effect. The prior used for additive marker effect $\beta_{k}$ has a mixture of distribution given below (Gianola *et al.* 2009).

(11) $$\beta_{k}|\pi ∼\mathrm{IID}\left\{\begin{array}{c} 0\mathrm{with}\mathrm{prob}\pi ,\\ t\left(0,v,S_{\beta }^{2}\right)\mathrm{with}\mathrm{prob}\left(1-\pi \right) \end{array}\right\},k=1,2,\ldots ,p$$

The Bayes B model assigns a different variance to each marker. The *π* value in Bayes B is treated as unknown and is specified from the data (Gianola 2013) with $\pi ∼\mathrm{Beta}(p_{0},\pi_{0})$, where $p_{0}$ denotes to the number of counts (priors successes plus prior failures) and $\pi_{0}$ is markers having null effects with $\pi_{0}\in [0,1]$.

We were also interested in how marker effects on fusiform rust disease incidence vary by controlling the level of markers having null effects (π_0_), a method called Bayes Cπ (Habier *et al.* 2011). In Bayes Cπ, the value of *π* is unknown. The effect of each SNP comes from a mixture of multivariate student’s *t*-distributions (Habier *et al.* 2011). We tested eight different prior distributions for the percent of markers with null effect (π_0_ = 0.99–0.10) to assess the completely polygenic *vs* oligogenic inheritance for the trait. All genomic prediction models were fit using the R package BGLR (Pérez and de Los Campos 2014), using 100,000 iterations, 20,000 burn-in, and thinning length of 50 iterations. Model convergence was visually assessed with trace plots created from samples of residual variance. No erratic behaviors were detected in trace plots of residuals indicating stable convergence of the models (Supplementary Figure S3).

#### Cross-validation

Ridge regression and Bayes B models provided better fit statistics (DIC and predictive ability) for fusiform rust disease compared to Bayes Cπ model. The following cross-validation (CV) scenarios were implemented to test the predictive ability of all SNP markers using ridge regression and Bayes B models. A summary of the cross-validation scenarios (sampling) is given in Supplementary Table S1.

1. *Random-CV:* A fivefold cross-validation scenario was performed by randomly selecting 80% (578 clonal varieties) of the population as the training set and the remaining 20% (145 clonal varieties) as the validation set. The random sampling was repeated 30 times. In this cross-validation scenario, the family structure and the relatedness were ignored.
2. *Family-CV:* Four full-sib families out of five were used as the training set, and the remaining one full-sib family was used as the validation set. The size of the training and validation sets differed for each *family-CV* scenario. Depending on the full-sib families included, the training set ranged between 503 and 677 clonal varieties while the testing set ranged between 46 and 224 clonal varieties.
3. *Within-family-CV:* In this scenario, 80% of each full-sib family was randomly selected to create the training set. The training population size was about the same (579 clonal varieties) as *random-CV* scenario. The validation sets (the remaining 20% of each full-sib family) had a range of 9–45 clonal varieties, depending on the family size. Within each family, the random sampling was repeated 30 times.

Predictive ability between the training and validation sets was estimated as $r_{pa}=r(y_{vs},{\hat{g}}_{vs})$ (Isik *et al.* 2016), where $y_{vs}$ denotes the best linear unbiased estimates (BLUE) and ${\hat{g}}_{vs}$ is genomic estimated breeding value (GEBV) from the validation set (vs). We also calculated the rank correlations $r_{\mathrm{rank}}=(y_{vs},{\hat{g}}_{vs})$ between GEBV and BLUE for each run. The mean estimates of the predictive ability and rank correlations were reported. Mean squared error (MSE) for each fold was calculated to assess the model fit.

#### Validation of QTL effects (GWAS)

Bayes B and Bayes Cπ models revealed three SNP loci with large effects. The following generalized linear mixed model was fit to estimate allelic substitution effect for significant loci detected in the Bayesian whole genome regression models. where, $b_{i}$ is the vector of additive marker *i-th* effect, $X_{i}$ is the design matrix of the fixed covariate marker with elements −1, 0, and 1. The p is effect of random experimental design effects. a is the additive (polygenic) effect of clonal variety associated with the additive genomic relationship matrix. Other terms were previously defined. Subsets of the models containing one, two, and all three QTL effects with or without polygenic effects were compared using average standard error of difference between predictions and average prediction error variance (Isik *et al.* 2017). The odds ratios of SNP loci genotypes (AA, AB, and BB) for fusiform rust disease outcome and percent variance explained by three QTL were estimated. The effect (odds ratio) of favorable allele dosage across all loci was estimated by constructing the haplotypes from the three SNP loci.

$\eta =\log\left(\frac{p}{1-p}\right)=\mu +s+\sum_{i=1}^{n}{b_{i}X}_{i}+p+a+e$ (12)

## Results

### Genetic parameter estimates

**Figure 1:**
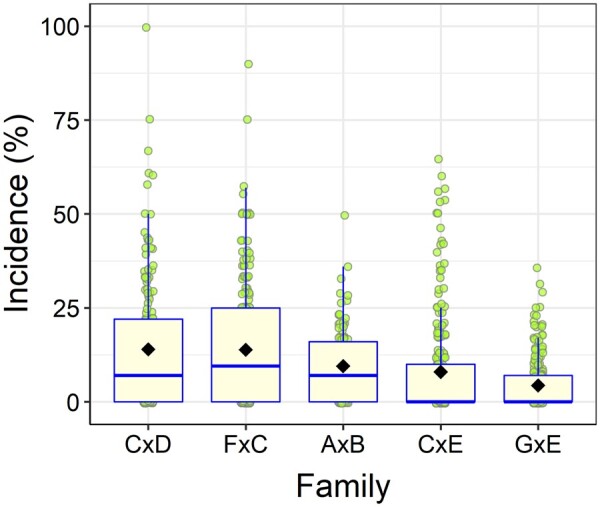
Boxplot showing incidence of fusiform rust disease for the genotyped full-sib families. Horizontal line in the middle of the box is the median and black diamond inside each box represents the mean. The boxes are in the interquartile range (50% of the data). Green circles denote individual clonal varieties.

Fusiform rust disease incidence had an overall mean of 9.7% in the studied population. The five genotyped full-sib families had a mean of 4 to 14% disease incidence. The mean incidence varied for clonal varieties within the families, ranging between 0 and 100% (Figure 1).

The additive genetic variance estimate was significant based on log-likelihood ratio tests, while the nonadditive genetic variance estimate was negligible. The clone-mean narrow-sense and broad-sense heritability estimates were high (0.87 and 0.93, respectively). Genotype-by-environment interaction was negligible, as shown by the high additive genetic correlations between pairs of sites (*r_a_* = 0.95) (Table 1).

**Table 1:** Genetic parameter estimates for fusiform rust disease incidence estimated from linear mixed model with additive genetic relationship matrix

Parameter	Fusiform rust (SE)
Mean incidence %	9.70 (0.167)
Additive genetic variance ($\sigma_{a}^{2}$)	2.49 (0.450)
Nonadditive genetic variance ($\sigma_{n}^{2}$)	0.16 (0.201)
Clone-mean narrow-sense heritability ($h_{\bar{c}}^{2}$)	0.87 (0.079)
Clone-mean broad-sense heritability ($H_{\bar{c}}^{2}$)	0.93 (0.006)
Additive genetic correlation between pairs of sites ($r_{a}$)	0.95 (0.023)

Standard errors (SE) of the estimates are provided in parentheses. Fusiform rust disease appears to be under strong additive genetic control.

### Clustering and genetic relationships

**Figure 2:**
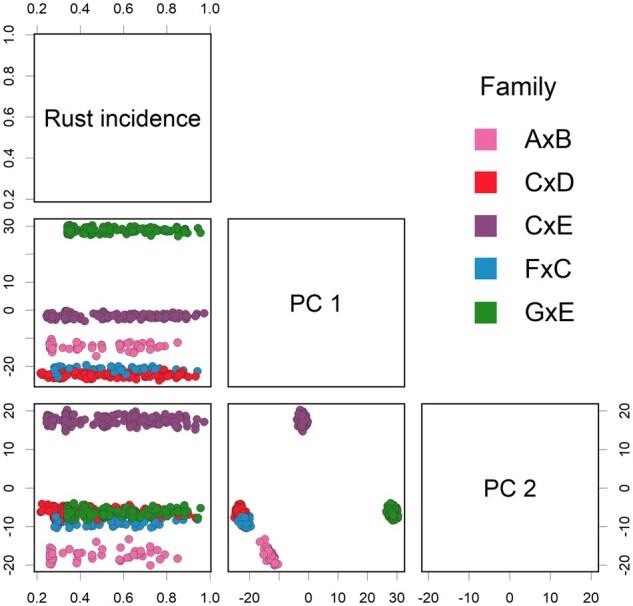
Spectral decomposition of genomic relationship among clonal varieties based on SNP markers across five full-sib families. The principal component analysis of marker data clustered clonal varieties into five distinct groups corresponding with the genomic relationships (full-sib families). The first and second principal components were plotted against the incidence of fusiform rust disease (transformed BLUEs). The circles represent individual genotypes and colors represent full-sib families.

**Figure 3:**
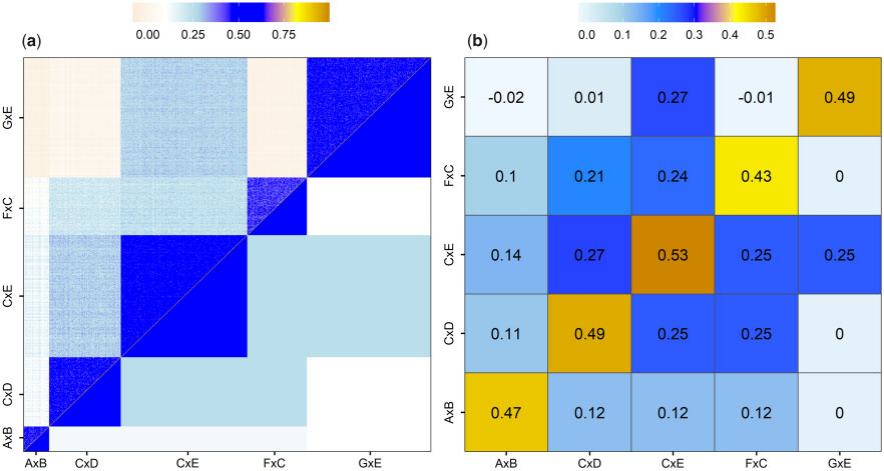
Heatmaps showing realized genomic (diagonal + upper diagonal) and expected additive genetic (lower diagonal) relationships calculated from SNP markers and pedigree, respectively. (A) Genetic relationships between 723 clonal varieties. The five dark blue blocks in the diagonal of the matrix correspond to full-sib families. (B) Average relationships within families (orange blocks in the diagonal of the matrix) and between five full-sib families. The realized genomic relationships (above diagonal) were slightly lower for some of the families and family pairs compared to expected genetic relationships (below diagonal).

Principal component analysis of SNP marker data put 723 clonal varieties into five distinct clusters, each representing a full-sib family (Figure 2). The clusters remained distinct when the first and second principal components were plotted against the incidence of fusiform rust disease (Figure 2). Families CxD and FxC formed a cluster, differentiating from the other three families. The clustering corresponded well with the expected genetic and realized genomic relationships. The five families were clearly visible as sub blocks in the diagonal of the matrix (Figure 3A). Overall, the realized genomic relationship agreed with the expected genetic relationships. Mean expected and realized genetic relationships for individual families and family pairs are presented in Figure 3B. The mean realized genomic relationship coefficients for the family pairs were slightly lower than expected genetic relationships derived from pedigree.

### QTL effects

**Figure 4:**
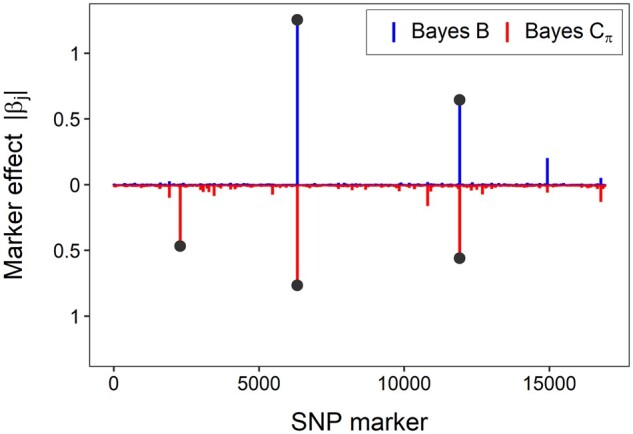
Marker effects in *y*-axis (absolute value) for fusiform rust disease from Bayes B (blue) and Bayes Cπ (red) models. The π is the proportion of nonnull effect markers with π = 0.01. Bayesian models revealed three SNP loci with large effects on fusiform rust disease.

Bayesian models (Bayes B and Bayes Cπ, π_0_ ≥ 0.90) detected three SNP loci (SNP6347, SNP11965, and SNP1920) with large effects on fusiform rust disease incidence (Figure 4). No major QTL effects were observed by Bayesian ridge regression and Bayes Cπ models with π_0_ < 0.90. The results of Bayes Cπ models with π_0_ < 0.90 were comparable to the Bayesian ridge regression model. The absolute effect of the three SNP loci identified by the Bayesian models was greater than 0.5. The effects were clearly visible when plotted (Figure 4). These three loci explained about 54% of genetic variation in disease incidence. Weak LD (≤0.17) between pairs of loci (Supplementary Figure S2), suggested that they are likely unlinked. We could not map these loci to the current version of *P. taeda* reference genome v2.01, because the markers used in this study were called using the *P. taeda* reference genome v1.0, a highly fragmented draft genome assembly with more than 15 million scaffolds (Zimin *et al.* 2014).

We validated the effects of three QTL as fixed covariates in GWAS. All three SNPs were significant at the α = 0.05 level (*F* tests probability values <0.0001). The model with three SNP loci as fixed covariates while accounting for polygenic effects had a lower average standard error of pair-wise prediction (1.02) and prediction error variance (1.04) compared to the model with polygenic effects only (1.37 and 1.86, respectively).

**Figure 5:**
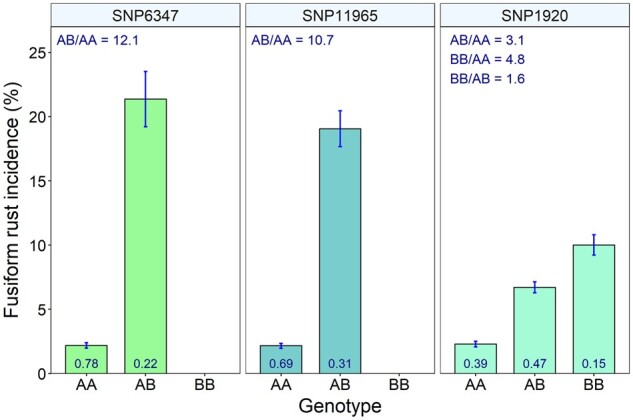
Mean fusiform rust disease incidence (%) with standard errors of genotypic classes (AA, AB, and BB) for the three loci detected by the Bayesian models. The odds ratio for the genotypic classes of the three SNPs are provided at the top left of the figures. Genotype frequencies are provided at the bottom of the bars. The odds of fusiform rust disease were significantly higher in BB and AB genotypes compared to AA.

The incidence of fusiform rust disease substantially decreased with the dosage of the A allele (Figures 5). Homozygous major genotypes AA showed significantly lower rust disease incidence (2%) compared to the heterozygous AB (∼20%) genotypes. The odds of fusiform rust disease incidence for heterozygous (AB) individuals were 10.7 and 12.1 times greater than the homozygous (AA) individuals for the two loci SNP6347 and SNP11965. Minor allele homozygous genotypes were not segregating for these two SNP loci in the population (Supplementary Table S2).

The odds of disease outcome for the haplotype with three B dosage (AAABBB) was 16 times higher than the odds of outcome for the homozygous haplotype AAAAAA (Supplementary Figure S4). This homozygous haplotype comprised 28% (203 individuals) of the population. More than half (114) of these individuals were in family GxE and the rest were in families CxE (66) and CxD (23). Family GxE had the lowest incidence of fusiform rust disease in the population (Figure 1).

### Predictive ability of markers

The summary of predictive ability of SNP markers for fusiform rust incidence, rank correlations, and model mean squared error statistics are given in Tables 2–4. Among three CV scenarios, *Random-CV* resulted in the highest average predictive ability estimates for Bayes B (0.71) and Bayesian ridge regression (0.76). This scenario also produced higher rank correlations and smaller mean squared error variance (better) compared to other CV scenarios (Table 2).

**Table 2:** Mean (range) predictive ability estimates, rank correlations, and mean squared error from Bayes B and Bayesian ridge regression models for the *random-CV*

Model	Predictive ability	Rank correlation	MS error
Bayes B	0.76 (0.66–0.84)	0.74 (0.63–0.83)	13.5 (10.3–16.9)
Ridge regression	0.71 (0.67–0.76)	0.70 (0.64–0.74)	15.3 (13.7–17.8)

The training set size was 578 (80%), while the validation set was 145.

**Table 3:** Predictive ability estimates, rank correlations and mean squared error from Bayes B and Bayesian ridge regression models for the *family-CV*

Model	Validation set	Predictive ability	Rank correlation	MS error
	Family (size)			
Bayes B	AxB (46)	0.04	0.03	23.1
	CxD (127)	0.10	0.05	22.0
	CxE (224)	0.56	0.52	34.3
	FxC (106)	0.29	0.27	36.6
	GxE (220)	0.48	0.50	41.3
Ridge regression	AxB (46)	0.10	0.05	40.9
	CxD (127)	0.13	0.06	20.3
	CxE (224)	0.42	0.39	16.7
	FxC (106)	0.30	0.27	19.9
	GxE (220)	0.37	0.39	49.1

There was no random sampling in *family-CV* and only one estimate was provided. Four full-sib families out of five were used as the training set. The validation set size is provided within parenthesis after each family ID.

**Table 4:** Mean (range) predictive ability estimates, rank correlations, and mean squared error from BayesB and Bayesian ridge regression models for *within*-*family-CV*

Model	Validation set	Predictive ability	Rank correlation	MS error
	Family (size)			
Bayes B	AxB (9)	0.52 (0.13–0.78)	0.38 (–0.30 to 0.80)	11.9 (7.1–22.7)
	CxD (25)	0.66 (0.44–0.82)	0.68 (0.48–0.83)	11.4 (7.5–17.2)
	CxE (45)	0.56 (0.28–0.79)	0.48 (0.29–0.69)	13.7 (8.3–17.9)
	FxC (21)	0.41 (0.24–0.65)	0.42 (0.12–0.64)	19.1 (13.6–23.8)
	GxE (44)	0.54 (0.39–0.68)	0.55 (0.43–0.72)	13.7 (8.9–17.8)
Ridge regression	AxB (9)	0.60 (0.10–0.80)	0.43 (−0.37 to 0.87)	11.8 (3.8–21.6)
	CxD (25)	0.53 (0.29–0.72)	0.59 (0.32–0.78)	14.1 (10.5–18.5)
	CxE (45)	0.50 (0.32–0.70)	0.46 (0.16–0.71)	14.8 (11.8–18.0)
	FxC (21)	0.41 (−0.05 to 0.75)	0.43 (−0.04 to 0.66)	18.2 (12.4–25.1)
	GxE (44)	0.38 (0.14–0.52)	0.40 (0.20–0.54)	17.2 (12.6–24.0)

The training sets were formed by randomly sampling 80% of each family and combining them (579 clonal varieties) to predict validation sets (20% of the family) within families. The validation set size (20%) for each family is given within parenthesis.

*Family-CV* scenarios produced low to moderate predictive ability estimates, ranging between 0.04 and 0.56 (Table 3). Using family CxE as the validation set produced the highest predictive ability among *family-CV* scenarios because of its relatively strong genetic relatedness with the training population (Figure 3B). The trend was similar for rank correlations and mean squared error variance. Bayes B and Bayesian ridge regression models varied for some families, but they produced similar statistics for the others.

*Within-family-CV* predictive ability estimates, with a range of 0.38–0.66, were considerably higher than the estimates for *family-CV* scenario (Table 4). Rank correlations had a similar range as the predictive ability estimates. Like *random-CV*, this CV had smaller mean squared error statistics compared to *family-CV* scenario. Bayes B and Bayesian ridge regression models produced similar predictive ability estimates.

## Discussion

### QTL effects on disease outcome

Fusiform rust disease incidence caused by the endemic fungus *C.* *quercuum* f. sp*. fusiforme* in *P. taeda* is under strong genetic control as evident from high clone mean heritability estimates, large QTL effects, and high genomic prediction accuracies. Bayesian interpretation of whole-genome regression models found large QTL segregating in the population suggesting an oligogenic inheritance. The effects of the three SNP loci were substantial, explaining about 54% of the total variance providing supporting evidence for major QTLs affecting the trait. Previous studies also showed that Bayesian models were effective in detecting markers with large effects and predicting fusiform rust disease incidence with high accuracy in *P. taeda* (Resende *et al.* 2012; Quesada *et al.* 2014). The three QTL detected in this study behaved additively. As the dosage of the favorable allele increased, the odds of disease outcome decreased (Figure 5, Supplementary Figure S4). In another *P. taeda* breeding population, Quesada *et al.* (2014) observed lower rust disease incidence for a homozygous major allele genotype. The authors used a different set of SNP markers discovered via the Illumina Infinium platform. The population of *P. taeda* used in this study has been under selection for fusiform rust disease resistance by ArborGen Inc. Selection for resistant genotypes in multiple breeding cycles may have resulted in a higher frequency of QTLs controlling the disease outcome in the population.

The three SNPs with large effects on the trait had weak pairwise linkage disequilibrium values, suggesting that they might be on different linkage groups. We did not have data to identify the position of the three major SNP loci on *P. taeda* reference genome v2.01 or sequence details of loci in both flanking regions. These SNP markers were called using the first draft of the *P. taeda* reference genome v1.0 (Neale *et al.* 2014), which was highly fragmented with ∼15 M scaffolds. To consider using these three QTL in *P. taeda* breeding program for selection of disease resistance genotypes, they need to be validated in an independent study. Understanding their sequence variation and location in *P. taeda* genome are important steps to develop DNA assays for marker-aided selection in *P. taeda* breeding. Estimation of allele substitution effect and fitting loci with large effects may increase prediction accuracies in estimation of genomic breeding values of disease incidence in *P. taeda*. In estimation of allele substitution effect, scaling might be an important factor for alleles with low frequencies (Bouwman *et al.* 2017). Low minor allele frequency loci (<0.01) are more common in conifers because of their large genome, large effective population size, and very recent breeding history (Neale *et al.* 2014).

### Genomic prediction within families

Genomic selection for fusiform rust disease in *P. taeda* breeding is promising. The random cross-validation scenario produced the highest prediction accuracies. It was closely followed by the random sampling within families to establish a training set and predicting the GEBV of the remaining 20% of progeny in each family. Using four families as the training set to predict GEBV of a remaining family produced low prediction accuracies. Studies on rubber tree yield and Sitka spruce growth reported 0.17–0.60 within-family prediction accuracies (Fuentes-Utrilla *et al.* 2017; Cros *et al.* 2019). Similar within-family prediction accuracy results were also reported in wheat and triticale *Fusarium* head blight disease incidence assessed as a binary trait (Würschum *et al.* 2017; Herter *et al.* 2019).

Genetic relatedness between the training and validation is an important factor on the prediction accuracy of genomic estimated breeding values (Solberg *et al.* 2008; Misztal 2011; Würschum *et al.* 2017). In this study, we found low predictive ability of markers when the genetic relatedness between the training and the validation sets were weak (*family-CV*). These results agree with previously published studies, reporting that among-family prediction accuracies were low due to weak genetic relatedness between training and validation sets (Wang *et al.* 2014; Han *et al.* 2016; Toro *et al.* 2017). Marker-trait phase might be different in a genetically independent population and the linkages between the QTLs and maker loci may not hold (Isik 2014). This is not surprising since, with genomic selection, we are likely tracing chromosome haplotypes segregating in the pedigree and thus capturing genetic relationships better rather than capturing the LD (Misztal 2011). In a simulation study on GS in plant breeding, about 1000 individuals were recommended for the training set if validation set is closely genetically related to the training set but 5–20 times more individuals were suggested if the training set is not closely linked to the validation set (Hickey *et al.* 2014). Genomic selection can be performed for within-family selection in *P. taeda* to capture Mendelian segregation effects if the training set for the model is genetically connected to the validation set.

One of the limitations of this study was small sample size, for some families, which hindered testing the predictive ability of markers within such families (small training sets). Hence, we used 80% of individuals from other families to make the training population size comparable to the *random-CV* scenario. Low overall fusiform rust disease incidence (10%) was another caveat of the study. Low incidence of fusiform rust disease is not surprising and has been widely reported from cloned progeny tests (Foster and Anderson 1989; Frampton *et al.* 2000; Isik *et al.* 2003; Shalizi *et al.* 2020). Compared with seedling progeny, vegetative propagules of *P. taeda* are resistant to fusiform rust disease in field trials (Frampton *et al.* 2000). The mean of a binomial distribution is related to its variance and lower disease incidence would have smaller variance among genotypes, which might bias estimates downward (King and Zeng 2001).

## Conclusions

We identified three SNP loci with a large effect on fusiform rust disease outcome in a set of clonally replicated full-sib families of *P. taeda*. These markers should be further validated in an independent study to be considered for marker-assisted selection. Within-family genomic selection for fusiform rust incidence could be effective in *P. taeda* breeding. A training population that is genetically linked to the prediction population is recommended for predicting individuals within families. Bayesian models could capture loci with large effects in fusiform rust disease in southern pines and should be preferred over the whole genome regression models with uniform distribution.

## Data Availability

All data used in this manuscript are available in supplemental materials uploaded on figshare https://doi.org/10.25387/g3.13589606. File Shalizi-pedigree.csv includes pedigree of all clonal varieties used to estimate variance components and breeding values. File Shalizi-phenotype.csv includes raw phenotype data used to estimate variance components, breeding values, and adjusted phenotypes. File *Shalizi-SNPmarker-adjustedPhenotype.rda* includes adjusted phenotypes and SNP markers for 723 *P. taeda* clonal varieties used to estimate marker effects and predict genomic estimated breeding values.
